# Validity and Reliability of a Non-invasive Test to Assess Quadriceps and Hamstrings Strength in Athletes

**DOI:** 10.3389/fphys.2018.01702

**Published:** 2018-11-29

**Authors:** Davide Mondin, Julian A. Owen, Massimo Negro, Giuseppe D’Antona

**Affiliations:** ^1^School of Sport, Health and Exercise Sciences, Bangor University, Bangor, United Kingdom; ^2^CRIAMS-Sport Medicine Centre, University of Pavia, Voghera, Italy

**Keywords:** hamstring, strength, injury risk, isokinetic, team sports

## Abstract

Modifiable risk factors for hamstring injury include lack of strength, fatigue and muscle strength asymmetry. Assessing lower body strength in the field is problematic as “gold standard assessment” are expensive, non-portable and assessment is time-consuming. Therefore, the objective of this study was to examine the validity and reliability of an adapted aneroid sphygmomanometer test of hamstring and quadricep strength. In 14 active males (age 23.1 ± 2.5 years; height 180.9 ± 8.2 cm; weight 88.4 ± 8.5 kg). concurrent validity was assessed by comparing the adapted sphygmomanometer assessment at 30 and 90° of knee flexion to isokinetic dynamometry using Pearson product-moment correlation. The reliability of the adapted sphygmomanometer was assessed in 10 professional rugby players (age 21.5 ± 2.6 years; height 177.2 ± 5.8 cm; weight 92.7 ± 5.8 kg ) across two visits. Sphygmomanometer strength assessments of hamstring and quadriceps were associated with isokinetic measures (Quadricep: right, *r* = 0.386, 95% CI = 0.136–0.866, *p* < 0.05; left, *r* = 0.431, 95% CI = 0.193–0.880, *p* < 0.05), hamstring strength at 90° of knee flexion (Hamstring: right, *r* = 0.545, 95% CI = 0.342–0.912, *p* < 0.01; left, *r* = 0.643, 95% CI = 0.473–0.935, *p* < 0.001) and hamstring strength at 30° of knee flexion (right, *r* = 0.329, 95% CI = 0.062–0.846, *p* < 0.05; left, *r* = 0.387, 95% CI = 0.138–0.867, *p* < 0.05). However, the adapted test was not able to identify bilateral or hamstring to quadricep asymmetry. Test–retest reliability was high for most assessments (ICC range: 0.64–0.92), and SEM measures ranged between 5 and 12%, with the smallest change representing a change in strength ranging between 3 and 4%. In conclusion, an adapted sphygmomanometer test for hamstring and quadricep strength assessment was valid and reliable in assessing hamstring and quadricep strength but not bilateral or hamstring and quadricep asymmetry.

## Introduction

Hamstring strains are one of the most frequent non-contact injuries in sport, especially in those that involve repetitive bouts of maximal sprinting (Schache et al., 2011). In football, hamstring injuries account for 12% of total injuries (Woods et al., 2002), with incidence rates of 18% in competitive sprinters (Yeung et al., 2009) and 12% in American football players (Feeley et al., 2008) while in rugby codes hamstring injuries represent 15% of total injuries per season (Brooks et al., 2006). Non-modifiable risk factors for hamstring strain, include: older age, ethnicity and previous injury (Verrall et al., 2001; Schache et al., 2011). Whereas modifiable risk factors of hamstring injury include, lack of hamstring strength, hamstring fatigue, strength asymmetries between quadricep and hamstring, and between left and right legs (Croisier et al., 2002; McCall et al., 2014, 2015). Consequently, hamstring and quadriceps strength should be screened regularly in team sport athletes to identify those at an increased risk of hamstring injury (Schache et al., 2011).

Isokinetic dynamometry is considered the “gold standard” screening tool for the assessment of hamstring and quadriceps strength (Harding et al., 2017). However, its use is limited in applied settings, due to high cost of the device, lack of portability and time needed to complete assessments (Opar et al., 2013). Therefore, research investigating the efficacy of alternative tests to monitor hamstring and quadriceps strength is warranted. Tests that are appropriate to the applied setting include; measures of eccentric bilateral strength such as the nordic hamstring test and assessments of explosive strength such as vertical jump testing. Although these tests are reliable, there is an increased risk of injury with eccentric loading (Brown and Weir, 2001; McCall et al., 2015). One repetition maximum (1 RM) tests represent a valid means to evaluate leg strength but precludes the assessment of specific muscle imbalances or bilateral asymmetry (Verdijk et al., 2009).

A potential solution is the use of isometric tests for injury screening as they pose a reduce likelihood of injury during assessment (Brown and Weir, 2001; McCall et al., 2015). Previous research has adopted the use of hand-held dynamometers as substitute measures of strength during hip adduction, abduction and knee flexion, and these proxy measures compare favorably with isokinetic assessments, but show limitations with respect to reliability (Bohannon, 1990; Piao et al., 2004; Stark et al., 2011; Thorborg et al., 2013; Souza et al., 2014). Others have examined the adapted use of a manual sphygmomanometer, whereby the aneroid manometer is used to give a value of pressure during various strength tests. This adaptation of a sphygmomanometer has demonstrated good validity and reliability for the assessment of muscle strength at 30 and 90° of knee flexion, and adductor strength, in clinical settings (Falla et al., 2004; Jull et al., 2008; Malliaras et al., 2009; Nevin and Delahunt, 2014). Nevertheless, research examining the reliability and the validity of these tests to assess strength of hamstring and quadriceps is limited (Helewa et al., 1993; Delgado et al., 2004; Martins et al., 2014; Souza et al., 2014), and much of this research has omitted comparison with more valid tests and been carried out in clinical populations meaning these findings cannot be generalized to athletic populations (Delgado et al., 2004; Martins et al., 2014; Souza et al., 2014).

The development of a non-invasive test utlising an adapted sphygmomanometer may provide a rapid, simple and cost effective method of assessing hamstring and quadriceps strength for use in field-based settings. Therefore, the aims of this study were, firstly, to evaluate the validity of an adapted sphygmomanometer test to evaluate hamstring and quadriceps strength and strength asymmetry at 30 and 90° of knee flexion, compared with isokinetic dynamometry and secondly, to evaluate the test–retest reliability in a group of professional rugby union players. We hypothesized that hamstring and quadricep strength assessment using this adapted sphygmomanometer test would be related to strength assessments of the same muscle groups using isokinetic dynamometry and consequently this adapted test would be suitable in detecting muscle strength asymmetries in field-based settings.

## Materials and Methods

### Participants

In this two-part study, 24 healthy male participants were recruited (age 22.5 ± 2.7 years; height 179.4 ± 7.3 cm; weight 90.2 ± 10.9 kg). For part-one, the validity and reproducibility of the adapted sphygmomanometer test for assessing hamstring and quadriceps strength was examined in 14 healthy male participants, free from lower limb injuries for the previous 12 weeks and actively and regularly participating in team sports (age 23.1 ± 2.5 years; height 180.9 ± 8.2 cm; weight 88.4 ± 8.5 kg ). For part-two, the test–retest reliability of the adapted sphygmomanometer test was examined in 10 male-professional rugby union players during the pre-season period (age 21.5 ± 2.6 years; height 177.2 ± 5.8 cm; weight 92.7 ± 5.8 kg). Participants were required to abstain from alcohol and unaccustomed exercise for 48 h before all experimental trials, and all subjects gave written informed consent before the study, which received local ethics committee approval.

### Study Design

**FIGURE 1:**
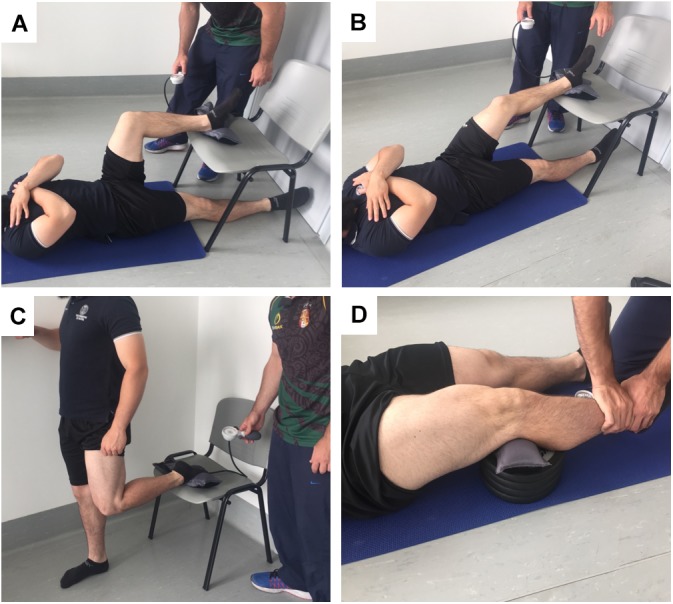
Orientation for the assessment of hamstring and quadricep strength using the adapted sphygmomanometer test. Panel **(A)** is quadricep strength assessment at 90° of knee flexion, panel **(B)** is quadricep strength at 30° of knee flexion, panel **(C)** is hamstring strength assessment at 90° of knee flexion, panel **(D)** is hamstring strength at 30° of knee flexion.

For part one of the study, and to investigate the concurrent validity of the adapted sphygmomanometer test to assess hamstring and quadricep strength, a comparison was made with isokinetic dynamometry. In a randomized design, concentric hamstring and quadricep strength was assessed by isokinetic dynamometry and strength measured with the adapted sphygmomanometer test. Following a thorough warm-up, participants performed three maximal contractions on each leg for each test, with the best performance from the three attempts recorded. The adapted sphygmomanometer test measured the maximal isometric strength of the quadriceps and hamstrings at 30° and 90° of knee flexion, with strength expressed in millimeters of mercury (mmHg) via the sphygmomanometer scale (for illustration see Figure 1). The choice of knee flexion angle was based on previous research showing the reliability of these isometric tests in assessing muscle strength in soccer players (McCall et al., 2015). The isokinetic assessment measured the peak torque (Nm) during knee flexion and extension at 60°s^-1^. Asymmetry between dominant and non-dominant legs for hamstring and quadricep strength was calculated for each test, in addition to hamstring to quadricep ratio for each leg.

The second part of the study examined the test–retest reliability of the adapted sphygmomanometer test, across two assessments visits. Participants were divided in two groups and assessed on two different days to meet the organizational needs of the rugby team. On the two visits, hamstring and quadricep strength was assessed using the adapted sphygmomanometer tests using the same protocol as used in part one. Each visit was separated by 1 week to avoid relevant strength differences caused by training adaptation during preseason (Hopkins, 2000). To ensure the necessary recovery time before the visits athletes were asked to avoid intense training and activity for 2 days before each assessment, this was ensured by timing the testing sessions in-line with the club’s weekly 2-day rest periods (McArdle et al., 2010).

### Protocol

#### Isokinetic Dynamometery

Participants performed a standardized 10 min warm-up on a cycle ergometer before each test (Monark 814e, Varberg, Sweden), consisting of 7 min of cycling at 90 W, followed by 3 min at 120 W (McCall et al., 2015). Participants were then seated in the isokinetic dynamometer (HUMAC NORM Model 770, CSMi, MA, United States) at 85° of hip angle, and secured in position with stabilization straps (O’Sullivan et al., 2008). The axis of rotation of the dynamometer was aligned with the center of the lateral femoral condyle and the resistance pad at the end of the lever arm was positioned two centimeters proximal to the lateral malleolus (O’Sullivan et al., 2008). The isokinetic dynamometer assessed hamstring and quadriceps concentric peak torque in a range of motion (ROM) between 5° and 100° of knee flexion (O’Sullivan et al., 2008; Opar et al., 2013). In order to familiarize participants with the testing procedure, participants performed a specific warm-up that consisted of three sub-maximal concentric extension and flexion movements at 25, 50, and 75% of perceived maximal effort (Brown and Weir, 2001) with a speed of contraction set at 60°s^-1^ (Mjølsnes et al., 2004; Yeung et al., 2009). Following, the test protocol consisted of three sets of maximal contractions for each leg and each movement (knee extension, knee flexion) at angular velocities of 60°s^-1^ with 3 min rest between repetitions (Croisier et al., 2002, 2008; Mjølsnes et al., 2004; O’Sullivan et al., 2008; Opar et al., 2013; Tol et al., 2014). All torques were corrected for the effects of gravity, automatically from the device, so excluding the different weight of leg between the subjects. Participants received advance verbal instructions and encouragement to push as hard as possible, to facilitate maximal effort during testing, but did not receive any additional verbal or visual feedback during the test (O’Sullivan et al., 2008).

#### Adapted Sphygmomanometer Test

The test utilized a common aneroid sphygmomanometer (DS44 sphygmomanometer, Welch Allyn, NY, United States), as non-modified sphygmomanometers have been reported to be more valid and reliable than modified methods when assessing athletes (Martins et al., 2015). During assessment a similar movement-specific warm-up to that outlined for the isokinetic assessment was used, with three sub-maximal contractions for each type of contraction at 25, 50, and 75% of perceived maximal effort, separated by 30 s rest after each exertion. Hamstrings and quadriceps strength was assessed at 30° and 90° of knee flexion and consisted of three sets of maximal contractions for each leg and each movement, as previously described (Figure 1; Mjølsnes et al., 2004; Delgado et al., 2004; Schache et al., 2011; McCall et al., 2015). For hamstring isometric strength, participants were positioned supine with arm rested across the chest, and knees flexed at 90° or 30°, with the heel of one leg on the cuff, and with the opposite leg resting on the floor and extended (Figures 1A,B). Flexion of the knees was determined by the distance of hip from the chair and measured with a goniometer. For quadricep strength assessment at 90° the participant was standing upright with instep placed on the cuff of sphygmomanometer, the participant used a support for balance and the opposite knee was completely extended to minimize strength compensations (Figure 1C). For quadricep strength assessment at 30° the participant assumed the same position as that during the hamstring evaluation. The cuff of sphygmomanometer was positioned between a modifiable plinth and popliteal fossa of the leg (Figure 1D). During strength assessments, participants pushed their heel or instep into the sphygmomanometer cuff as hard as possible without lifting the buttocks off of the floor or compensating in any other fashion. The contraction was held for 5 s, and the peak pressure was recorded (?; Martins et al., 2015). Every contraction was separated by 30 s rest period to allow adequate recovery between trials (Brown and Weir, 2001). As with the isokinetic assessments, participants received advance verbal instructions and encouragement to push as hard as possible, to facilitate maximal effort during testing, but did not receive any additional verbal or visual feedback during the test.

#### Calibration

Based on pilot data collected from seven participants, the sphygmomanometer cuff was inflated to 20 mmHg before placing the participant’s foot on the cuff for each of the assessments. Calibration of the cuff was carried out using weighted plates balanced on the pre-inflated cuff, that would elicit cuff pressures in the range observed during pilot testing. Standard weighted plates (40 and 65 kg) were used to calibrate the sphygmomanometer cuff to ensure acceptable limits of precision. The coefficient of variation for repeated measurements was 0.8% at the lower end of the pressure range (200–204 mmHg for 40 kg weight) and 1.0% at the higher end of the pressure range (282–290 mmHg for 65 kg weight).

### Data Analysis

Using a Pearson’s correlation sample size calculator (Hulley et al., 2013) and data from previously published studies which reported a correlation coefficient of 0.8 between a modified sphygmomanometer and a portable dynamometer, a sample size of *n* = 14 was calculated with power levels set at 0.05 and 0.8, respectively (Martins et al., 2015; Silva et al., 2015). A total of 21 participants were initially recruited, however, due to initial methodological issues the data analysis only included the values for 14 participants. For the second part, a similar sample size estimation was utilized using previously published data on the reliability of the modified and traditional sphygmomanometer test in healthy young (20 to 30 years old) population (Delgado et al., 2004; Souza et al., 2014). A coefficient factor of 0.75, guaranteeing a possible margin of error. The results suggested a total of at least 10 participants and two visits to demonstrate the reliability. The concurrent validity of the adapted sphygmomanometer to assess hamstring and quadricep strength and hamstring to quadricep ratio, was tested using Pearson’s product–moment correlation coefficient (*r*) with 95% confidence intervals (CI). To analyze the test–retest reliability from the two assessment sessions, the intraclass correlation coefficient (ICC) with 95% CI and Cronbach’s alpha were used. Absolute reliability was determined with the SE of measurement and smallest real difference (SRD). These were calculated using the following formulas: SE of measurement = SD √(1 - ICC), where SD is the mean SD of visit 1 and visit 2 to represent total measurement variability; and SRD = 1.962×√(SE). The SE of measurement and SRD were also expressed as a percentage of the group mean for both test sessions for each of the variables. Finally, when analyzing the reproducibility of the adapted sphygmomanometer to assess strength over three consecutive attempts, the coefficient of variation (CV) was used. The level of significance was set at 0.05. All calculations were performed using Statistical Package for Social Sciences for Mac OSX 10.7 + (Version 22.0, SPSS Inc., Chicago, IL, United States).

## Results

### Concurrent Validity

**FIGURE 2:**
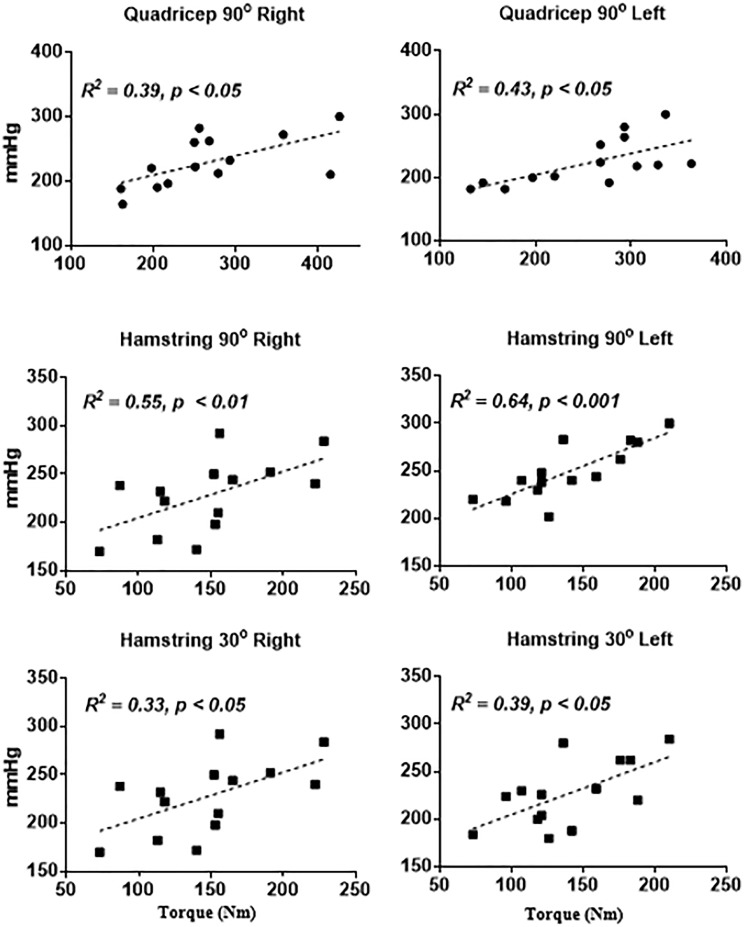
Relationship between sphygmomanometer tests and isokinetic dynamometry (x-axis). sphygmomanometer reading are shown on the x-axis and expressed in mmHg.

At 30° of knee flexion, quadricep strength assessments were not successful as in 9 of the 14 participants pressure recorded via the sphygmomanometer exceeded the readings on the scale. For the remaining dataset there was a positive correlation between the isokinetic dynamometer and adapted sphygmomanometer for the measurement of quadricep strength at 90° of knee flexion (Quadricep: right, *r* = 0.386, 95% CI = 0.136–0.866, *p* < 0.05; left, *r* = 0.431, 95% CI = 0.193–0.880, *p* < 0.05), hamstring strength at 90° of knee flexion (Hamstring: right, *r* = 0.545, 95% CI = 0.342–0.912, *p* < 0.01; left, *r* = 0.643, 95% CI = 0.473–0.935, *p* < 0.001) and hamstring strength at 30° of knee flexion (right, *r* = 0.329, 95% CI = 0.062–0.846, *p* < 0.05; left, *r* = 0.387, 95% CI = 0.138–0.867, *p* < 0.05) (see Figure 2).

### Measurement of Strength Asymmetry

When analyzing the efficacy of the adapted sphygmomanometer test to detect strength asymmetries between dominant and non-dominant legs, no relationship was found between tests at either 30 or 90° of knee flexion compared with isokinetic assessments (Hamstring asymmetry 30°, *r* = 0.371, 95% CI = -0.199–0.753, *p* = 0.19; Hamstring asymmetry 90°, *r* = 0.405, 95% CI = -0.160–0.770, *p* = 0.15; Quadricep asymmetry 90°, *r* = -0.499, 95% CI = -0.815–0.042, *p* = 0.07). There was also no correlation in hamstring to quadricep ratio at 90° of knee flexion between the tests for either right (H:Q right, *r* = 0.488, 95% CI = -0.057–0.809, *p* = 0.08) or left legs (H:Q left, *r* = 0.244, 95% CI = -0.329–0.686, *p* = 0.40).

### Reliability and Reproducibility

The intra-class correlation showed a high test–retest reliability for assessments of hamstring and quadricep strength using the adapted sphygmomanometer, except for right quadricep strength assessment at 90° of knee flexion (Table 1). Finally, when analyzing the reproducibility of the adapted sphygmomanometer tests for the measurement of 3 consecutive trials, similar coefficients of variation were observed in comparison to those obtained with the isokinetic assessments (Sphygmomanometer test, quadricep 90° right: CV = 4.6% ± 2.7; quadricep 90° left: CV = 7.5% ± 4.9; hamstring 90° right: CV = 3.8% ± 2.9; hamstring 90° left: CV = 3.4% ± 2.4; hamstring 30° right: CV = 5.1% ± 4.5; hamstring 30° left: CV = 5.9% ± 8.2; vs. Isokinetic assessment, quadricep right: CV = 5.7% ± 4.5; quadricep left: CV = 4.7% ± 3.0; hamstring right: CV = 9.2% ± 6.4; hamstring left: CV = 9.6% ± 8.7).

**Table 1:** Test–retest reliability of the adapted sphygmomanometer tests between two separate assessment visits.

Assessment	ICC (95% CI)	SEM (mmHg)	SEM (%)	SRD (mmHg)	SRD (%)
Quad 90° Right	0.64 (-0.28–0.91)	10.43	4.28	8.95	3.68
Quad 90° Left	0.81 (0.21–0.95)^∗^	11.67	5.02	9.47	4.07
Ham 90° Right	0.83 (0.30–0.96)^∗∗^	8.53	3.55	8.10	3.37
Ham 90° Left	0.87 (0.45–0.97)^∗∗^	6.75	2.80	7.20	2.98
Ham 30° Right	0.92 (0.69–0.98)^∗∗^	5.88	2.59	6.72	2.96
Ham 30° Left	0.87 (0.48–0.97)^∗∗^	6.36	2.85	6.99	3.13


Quad, quadriceps; Ham, hamstrings; ICC, intraclass correlation coefficient; SEM, standard error of measurement; SRD, smallest real difference. ^*^P < 0.05, ^**^P < 0.01 between the two assessment visits.

## Discussion

The main findings from this study were that adapted the sphygmomanometer test was valid in measuring hamstring and quadricep strength compared to measures of isokinetic concentric strength at 60°s^-1^. Specifically, data showed an association between the sphygmomanometer derived pressures at 30 and 90° of knee flexion and isokinetic strength measures as revealed by Pearson’s product-moment correlation coefficient. Despite associations between isokinetic measures and hamstring strength at 30 and 90° and quadricep strength at 90° of knee flexion, the sphygmomanometer test was not applicable to assess quadricep strength at 30° of knee flexion, as 9 out of the 14 participants recorded pressures beyond the measurement capability of the sphygmomanometer. The sphygmomanometer tests were also shown to be reliable across two separate assessment visits, as shown by the high intraclass correlation coefficients. Furthermore, when testing the reproducibility of the adapted sphygmomanometer tests across 3 separate trials for each participant, similar coefficients of variation compared with isokinetic dynamometry measures were observed (about 3–8%). Lastly, and despite the clear association between measures obtained from the adapted sphygmomanometer tests and isokinetic dynamometry for both hamstring and quadricep strength, the adapted sphygmomanometer was not valid to identify strength asymmetries between dominant and non-dominant legs or between hamstring and quadricep muscle groups. Therefore, our hypotheses are partially accepted and taken together, these results demonstrate that an adapted sphygmomanometer test for strength can be used to obtain valid and reliable measures of quadricep and particularly hamstring strength in the absence of costly laboratory equipment.

To our knowledge this is the first study to investigate the validity and reliability of a traditional aneroid sphygmomanometer to test hamstring and quadriceps strength in an athletic population, and the only study that compared the results of the sphygmomanometer test with the isokinetic dynamometery, that represents the gold standard for hamstring and quadriceps strength evaluation and for hamstring injury prediction (Harding et al., 2017). The findings from this study builds on a previous case study which investigated the application of the adapted sphygmomanometer test at 90° of knee flexion hamstring for prediction of hamstring injury risk (Schache et al., 2011). The results of this study are also in-line with a previous study investigating the reliability of non-invasive isometric hamstring strength assessments at 30 and 90° of knee flexion using a force platform (McCall et al., 2015). Indeed, the test–retest reliability indicated by the intraclass correlation coefficient derived from our study for hamstring strength assessments were similar to those reported in this study (Hamstring strength 30° knee flexion, ICC range = 0.86–0.93 vs. 0.87–0.92; Hamstring strength 90° knee flexion, ICC range = 0.88–0.98 vs. 0.82–0.87). In this study, the researchers found that the adapted force platform was sensitive to distinguish asymmetry in bilateral strength. However, our findings suggested that the adapted sphygmomanometer was not associated with isokinetic assessments of bilateral muscle strength asymmetry or hamstring to quadricep ratio, which may limit the use or applicability of this device. Another limitation of the device was the inability to consistently measure quadricep strength at 30° knee flexion, due to many participants exceeding the readings on the sphygmomanometer scale. Previous research has suggested the use of a modified sphygmomanometer to account for stronger participants (Souza et al., 2014). Nevertheless, it was possible to measure quadricep strength at 90° of knee flexion, and these measures were found to be associated with isokinetic measures and reliable within and between visits.

Hamstring strains are one of the most frequent non-contact injuries in sport, especially in those that involve repetitive bouts of maximal sprinting (Schache et al., 2011). Modifiable risk factors of hamstring injury include, lack of hamstring strength, hamstring fatigue, bilateral strength asymmetries and asymmetries between quadricep and hamstring (Croisier et al., 2002; McCall et al., 2014, 2015). Most methods for assessing hamstring and quadricep strength have limitations in field-based settings, including high cost, risk of injury during assessment, lack of portability and duration of assessment. In this study, we proposed a rapid and non-invasive proxy measure of hamstring and quadricep strength at 30 and 90° of knee flexion using an adapted sphygmomanometer. The findings of this study suggest that this novel test could be routinely used to assess changes in hamstring and quadricep strength in athletes. The advantage of this test is that the equipment is relatively inexpensive, the method is easy to administer, and measures are recorded rapidly, and therefore could form part of a routine athlete monitoring or screening program. Indeed, measures could be made immediately after or during a recovery period from even high intensity training. As the sphygmomanometer test requires only few minutes to test hamstring and quadriceps strength making it a useful test for potentially monitoring athlete strength changes during the training week. Future research should assess this device for measuring hamstring and quadricep strength on a larger sample size and examine the association between the adapted sphygmomanometer measures with other isometric dynamometry assessments that have proved useful in injury screening. In addition, it would be worthwhile to investigate whether the adapted sphygmomanometer test could identify decrements in muscle strength due to fatigue caused by training or competition. Furthermore, the device would be utilized to screen hamstring and quadricep strength longitudinally to determine if this test could predict hamstring injuries.

## Conclusion

The present findings showed that this simple, rapid, non-invasive test is valid and reliable enough to analyze quadriceps and hamstring strength in semi-professional and amateur athletes. The present test may represent a useful and practical field tool to determine strength variation of athletes and may be able to identify players who exhibit large reductions in hamstring strength.

## Ethics Statement

Ethical approval for this study was received from the research ethics committee of the School of Sport, Health and Exercise Sciences, Bangor University.

## Author Contributions

DM performed the experiments. DM and JO conceived the original idea. JO, DM, MN, and GD wrote the paper. JO and DM analyzed data.

## Conflict of Interest Statement

The authors declare that the research was conducted in the absence of any commercial or financial relationships that could be construed as a potential conflict of interest.
